# Invasive Evaluation of Coronary Artery Disease in Severe Aortic Stenosis—A Narrative Review

**DOI:** 10.3390/jcm15145354

**Published:** 2026-07-08

**Authors:** Harsh V. Thakkar, Habib Samady, Brian Ko, Adam J. Brown

**Affiliations:** 1 Victorian Heart Institute, Monash University, Victorian Heart Hospital, Clayton, VIC 3168, Australia; 2 Emory University School of Medicine, Emory University Hospitals, Atlanta, GA 30308, USA

**Keywords:** severe aortic stenosis, coronary physiology, FFR, CTFFR, TAVR

## Abstract

The coexistence of severe aortic stenosis (AS) and coronary artery disease (CAD) is common and presents important diagnostic and therapeutic challenges, particularly in patients being considered for transcatheter aortic valve replacement. Accurate assessment of coronary lesion significance in this setting is difficult because severe AS alters coronary haemodynamics, myocardial oxygen demand, microvascular function, and the balance between resting and hyperaemic flow. These changes may influence the interpretation of conventional physiological indices and complicate decisions regarding revascularisation. This narrative review summarises the pathophysiological interaction between severe AS and CAD and examines the contemporary evidence supporting invasive and non-invasive approaches to coronary assessment. We review the limitations and potential utility of fractional flow reserve, and non-hyperaemic pressure ratios, highlighting the frequent discordance observed between indices and the uncertainty regarding optimal thresholds in severe AS. Importantly, identification of physiologically significant lesions should be distinguished from evidence that revascularisation of these lesions improves clinical outcomes, as prospective outcome data remain limited. While recent trials support physiology-guided revascularisation in patients undergoing TAVR, outcome data remain linked primarily to conventional FFR thresholds rather than proposed AS-specific cutoffs. We also discuss emerging non-wire-based approaches, including quantitative flow ratio and computed tomography-derived fractional flow reserve, which may offer complementary value in selected patients. In addition, we examine the practical implications of coronary physiology for clinical decision-making before and after valve intervention, including the timing of percutaneous coronary intervention and the need to distinguish lesion-level diagnostic performance from evidence of clinical benefit. Current data suggest that no single modality is universally applicable and that assessment should be individualised according to lesion characteristics, clinical context, and procedural strategy. Proposed severe AS-specific thresholds for FFR and NHPR are derived from small predominantly observational studies, have not been prospectively validated against clinical outcomes and should be hypothesis-generating. A hybrid approach integrating angiographic, physiological, and computed tomography-based information may be most useful. Further prospective studies are needed to define optimal thresholds, validate management algorithms, and clarify whether physiology-guided strategies improve outcomes in severe AS.

## 1. Background

The prevalence of aortic stenosis (AS) has been exponentially increasing due to an ageing population [1]. Severe aortic stenosis (AS) is the commonest valvular pathology requiring intervention in developed countries [1]. Degenerative AS begins as subendothelial plaque-like deposits on the aortic side of the valve leaflet, described as aortic sclerosis, that progress over years to severe calcific aortic stenosis due to ongoing inflammation and calcium deposition [2]. The mechanistic process leading to aortic valve degeneration resembles atherosclerosis and elastocalcinosis [3]. Furthermore, compelling epidemiological data suggest that risk factors for AS and coronary artery disease (CAD) are similar [4]. Thus, it is not surprising that significant CAD is often present in up to 60% of patients with severe AS [5].

Historically, treatment for AS in its severe form has been surgical aortic valve replacement (SAVR). With the advent of transcatheter technologies, percutaneous options are now available [6,7]. Transcatheter aortic valve replacement (TAVR) is non-inferior to SAVR for patients across all surgical risk groups [8,9]. In keeping with the current evidence, the American and European cardiology societies have updated their guidelines to recommend TAVR to patients aged over 65 years with severe symptomatic AS, based on the patient/anatomical characteristics and Heart Team recommendations [10,11]. As a result, many younger patients with severe AS are being considered for TAVR. Hence, there is an increasing focus on accurate assessment and revascularisation of concomitant coronary artery disease (CAD), which would have been previously managed with a bypass grafting at time of SAVR. Current guidelines recommend percutaneous coronary intervention (PCI) for severe stenosis in the proximal coronary segments in major epicardial arteries, particularly if presenting with acute coronary syndrome, symptoms of angina or subocclusive segments prior to TAVR [12,13].

The use of non-invasive testing for ischaemia remains challenging in AS due to the difficulty in achieving a maximal workload due to the patient’s functional capacity. This is known to reduce the accuracy of stress echocardiography in detecting CAD [14]. There are overt difficulties in interpreting symptoms when AS and CAD coexist. Exercise or pharmacological testing are also contraindicated in severe symptomatic AS [15], due to risk of decompensation. This, therefore, leaves invasive angiography as the reference standard for the assessment of CAD in patients with severe symptomatic AS. The role of invasive metrics to assess CAD severity in AS, such as use of fractional flow reserve (FFR) and alternate non-hyperaemic pressure ratios (NHPRs) is currently explored and the validity of these measurements remains uncertain [16,17].

This review focuses specifically on the physiological assessment of CAD in the setting of severe AS. The aim is to address three key questions: why conventional physiological indices are confounded in severe AS, how invasive and non-invasive physiological tools perform in this context, and how clinicians can integrate these modalities into decision-making in their clinical practice. By critically appraising the current literature, a practical, expert opinion-based framework for assessment of CAD in patients undergoing TAVR will be presented.

## 2. Pathophysiology of Concomitant CAD and AS

Aortic stenosis and CAD, while distinct, share several pathophysiological pathways and risk factors. These commonalities contribute to the co-existence of these two pathologies in an individual. The initiating process involves lipid deposition, macrophage and T-cell infiltration leading to inflammation and basement membrane disruption. As the disease progresses, this leads to extracellular matrix formation, calcification, fibrosis, and endothelial dysfunction. Aortic stenosis differs from CAD due to a more extensive accumulation of extracellular calcification and the absence of smooth muscle cell proliferation [18,19,20]. Degenerative changes due to age and the prolonged exposure to mechanical stresses accentuate these processes in CAD and AS. Common risk factors such as age, male sex, diabetes, hypercholesterolemia, hypertension and chronic kidney disease further underscore the interconnectedness of these diseases [21]. The gradual and continuous advancement of AS, with clinical symptoms and cardiovascular events occurring with progressive reduction in leaflet mobility and development of left ventricular outflow obstruction [22], contrasts with the progression of CAD, which is characterised by periods of relative quiescence alternating with episodes of clinically apparent or even silent plaque rupture [23].

The current guidelines from the ESC and ACC/AHA recommending TAVR to patients above 65 years open a critical discussion on the management of CAD in severe AS. Furthermore, the prevalence of obstructive CAD in patients undergoing TAVR is higher than that typically reported in surgical series. This discrepancy is accounted for by the TAVR population being older and having a higher burden of medical comorbidities. Indeed, CAD was present in more than two-thirds of patients in a randomised trial of intermediate and high-risk TAVR candidates [6,7], and even a third of patients within a low-risk clinical trial [8]. For the functional assessment of CAD, FFR and NHPR are validated tools that help prognosticate and clarify the clinical significance of individual coronary lesions in stable coronary artery disease [24]. Nevertheless, their diagnostic accuracy and prognostic significance in patients with severe aortic stenosis is not precise and unknown respectively, highlighting the complexity and importance of this issue.

### Current Conundrums in Assessing Coronary Physiology in AS

**Figure 1:**
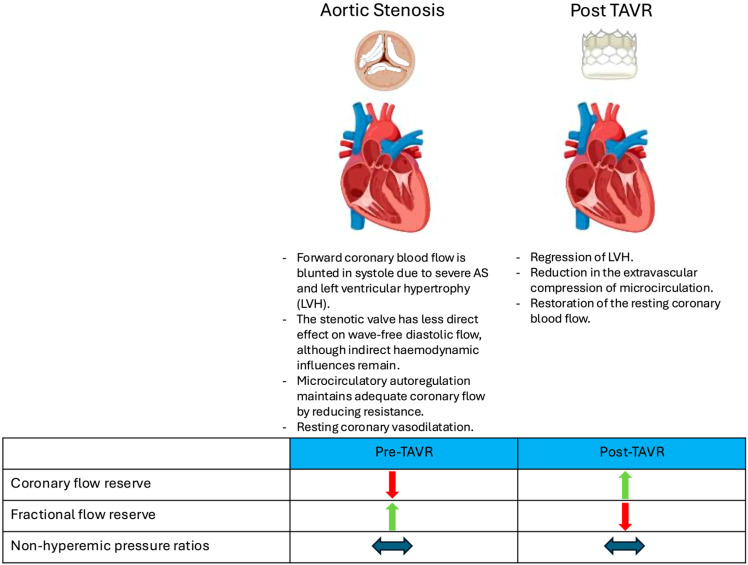
Changes in myocardial mass and epicardial physiology after aortic valve intervention.

A thorough understanding of the coronary pathophysiology for patients with severe aortic stenosis has potential to help clinicians negotiate the challenges in the cath lab when performing invasive measurements. In patients with severe AS, there is a significant increase in afterload, which increases LV wall stress. In response, there is subsequent myocardial hypertrophy to improve contractility and reduce wall stress [25]. These changes result in increased intracavity LV pressures, which reduces the diastolic gradient driving coronary perfusion. There is vasodilatation in response to muscle hypertrophy to maintain adequate perfusion at a microcirculatory level. The changes in the coronary perfusion pressures and the circulatory vasodilatory response are directly proportional to the extent of LV hypertrophy. There is no compensatory increase in the coronary microvasculature volume to match the extent of hypertrophied ventricle. The increased oxygen demand is not translated to improved perfusion in the coronary microvasculature due to the fixed elevated systolic wall stress and the reduced relative capillary density [26,27], creating a supply demand mismatch. With disease progression there is capillary rarefaction and perivascular fibrosis, further reducing the blood supply and leading to a graded decrease in blood supply to the LV wall. Thus, the subendocardial layer receives the least amount of blood, while the epicardial blood supply is relatively preserved [28]. The reversal of the typical endocardium/epicardium blood flow ratio at rest is pathognomonic for AS, which results in subendocardial ischaemia, apoptosis, and fibrosis [27,29] (Figure 1). The supply-demand mismatch, and structural and functional changes within the microvasculature exert adverse effects on the coronary microcirculation.

Coronary blood flow is phasic and occurs in both systole and diastole. During systole, the net flow is caused by emptying the left ventricle through the aortic valve into the aorta and the opposing compression forces from the contracting myocardium, which blunts the forward flow. In severe AS, the forward flow is reduced due to the obstruction caused by the stenotic valve and simultaneous compression of the microvasculature by the hypertrophied left ventricle opposing forward flow in the coronaries [30,31]. These mechanisms combine to result in reduced blood flow through the coronaries during systole. In diastole, during the wave-free period, flow occurs during the period when the myocardium is neither contracting nor actively relaxing [32]. During this period, the aortic valve is closed; thus, the aortic valve restriction, a systolic phenomenon, does not affect wave-free diastolic coronary flow. Microcirculatory autoregulation induces arteriolar vasodilatation to reduce microvascular resistance and preserve myocardial perfusion, thus causing a net increase in total resting myocardial blood flow and coronary velocity. The recruitment of microcirculatory reserve at rest to meet elevated metabolic demand diminishes the capacity for additional augmentation in times of stress or hyperaemia, resulting in reduced coronary flow reserve (CFR). With progressive severity of AS, the autoregulation ability reaches its limit, with no room to improve blood flow. These patients have increased coronary velocities and volumetric flow to match the increased resting demand, with insufficient reserve left to match any further increases in demand with exertion. This results in more potential for ischaemia than in a patient with normal LV mass or without severe AS. These physiological abnormalities occur along a continuum that parallels AS severity. Studies using invasive haemodynamic assessment, myocardial contrast echocardiography and positron emission tomography have consistently demonstrated progressive augmentation of resting coronary flow with a reduction in CFR as valve obstruction and LV hypertrophy worsen [16,26,27]. However, the available data have been derived from relatively small cohorts using different methodologies. Consequently, direct comparison between studies is challenging and a robust quantitative estimate of the degree of flow alteration across AS severity grades cannot currently be provided. Nevertheless, these studies consistently demonstrate progressive impairment in coronary vasodilatory reserve and increasing reliance on resting vasodilatation as AS severity advances.

Although FFR and NHPR are pressure-derived indices, their underlying physiological principles rely on the fact that pressure is proportional to coronary flow during their measurement. Therefore, any changes to flow directly affect their measurements. Thus, the value of FFR, which is measured over the whole cardiac cycle [33], is more affected in severe aortic stenosis than NHPR, especially the ones which are measured in the diastolic period [34], as diastolic physiology is less prone to changes from severe AS compared to systolic physiology [35]. Due to the blunting of the hyperaemic flow in severe AS, as described above, the FFR value will be underestimated, hence underestimating the severity of coronary stenosis. In comparison, certain NHPRs may be less affected by the presence of severe AS and their use may offer a theoretical advantage over FFR. It also does not require hyperaemia, which avoids the side effects of an adenosine infusion (bradycardia, heart block and hypotension). However, due to increased afterload, LV hypertrophy and increased oxygen demand, there is increased coronary blood flow during resting conditions, as described above, which may cause the NHPR to overestimate the coronary stenosis (Figure 1).

In summary, severe AS fundamentally alters coronary physiology through four interrelated mechanisms—increased resting coronary flow, impaired hyperaemic augmentation, reduced coronary flow reserve, and structural and microvascular dysfunction. These changes disrupt the relationship between coronary pressure and flow that underpins the pressure-derived indices, FFR and NHPRs. As a result, the conventional ischaemic thresholds may not be directly applicable, and the discordance between these indices should be expected rather than being interpreted as measurement error.

The following section summarises the published literature on invasive evaluation of coronary artery disease in severe aortic stenosis.

## 3. Invasive Evaluation of Coronary Lesions in Severe Aortic Stenosis

Current guidelines recommend hyperaemic and non-hyperaemic physiological indices to evaluate coronary lesions based on large clinical trials [33,34,36,37]. A summary of the trials looking at the use of hyperaemic and non-hyperaemic indices in severe AS is provided in Table 1.

Several consistent themes emerge from the available data. First discordance between FFR and NHPR is common, occurring in approximately 20–40% of the cases [48,50,52,54], and most frequently follows an FFR−/NHPR+ pattern. This pattern is physiologically plausible, reflecting the combination of reduced hyperaemic flow leading to falsely elevated FFR values and increased resting flow causing lower NHPR values. The clinical significance of the common FFR−/NHPR+ discordance pattern warrants consideration. Consequently, discordance should not be regarded as measurement error but rather as a physiological signal reflecting the competing effects of severe AS on resting and hyperaemic flow.

The clinical implications of this pattern remain uncertain. When FFR suggests lesion deferral, but NHPR suggests physiological significance, management should not rely on either index in isolation. Instead, clinicians should integrate lesion location, symptom burden, coronary anatomy, myocardial territory at risk and the anticipated timing of valve intervention. In lesions supplying large myocardial territories, particularly proximal epicardial vessels such as the left main coronary artery or proximal left anterior descending artery, additional assessment using complementary imaging modalities may be appropriate. Importantly, an abnormal NHPR alone should not be assumed to identify lesions that will benefit from revascularisation. Recent data from Jo et al. [51] suggest that an abnormal FFR value retained an association with adverse clinical outcomes, whereas NHPR appeared to classify a larger number of lesions as significant without a corresponding increase in prognostic discrimination. These findings support the concept that NHPR may overestimate lesion severity in some patients with severe AS because of elevated resting coronary flow. However, this study should be interpreted with caution, as it represents a single retrospective, observational dataset. Firstly, post hoc analysis of NHPR was performed using archived Pd/Pa curves. Secondly, there was a lack of predetermined criteria for physiological assessment, and no quantitative coronary angiography was performed, only visual assessment of stenosis severity. Lastly, there was no standardised decision-making for revascularisation for lesions with FFR ≤ 0.80; thus, some lesions were deferred based on operators’ decisions. Hence, the findings from this single study do not establish the superiority of one physiological index over another. Nevertheless, they highlight the distinction between diagnostic sensitivity for ischaemia and prognostic relevance. This study reinforces the need for outcome-based validation of any physiology-guided strategy in severe AS.

Second, optimal ischaemic thresholds for FFR and NHPR appear to differ from those established in stable CAD populations. Several studies suggested that a higher FFR threshold (approximately ≤0.83) [17,43,45] and a lower NHPR threshold (approximately ≤0.85) [16,17,35,43,44,45,46,55] may better reflect ischaemia in this cohort. However, these proposed thresholds are derived from relatively small studies focused predominantly on diagnostic performance and physiological concordance rather than clinical outcomes. They have not been prospectively validated in outcome-driven trials and should therefore be regarded as hypothesis-generating rather than definitive treatment thresholds. These findings reinforce the concept that the pressure-derived indices are highly dependent on underlying coronary flow conditions, which are significantly altered in severe AS.

Third, the effect of valve intervention on physiological indices is heterogeneous. While NHPR values demonstrate relative stability following TAVR [16,35,45,46,52,53] FFR often decreases [41,42,45,53], reflecting restoration of hyperaemic flow and relief of aortic outflow obstruction. However, these changes are heterogeneous and may depend on lesion severity, coronary flow patterns and degree of microvascular dysfunction.

Importantly, the prognostic relevance of these indices in severe AS remains incompletely defined. Most studies are observational, with small sample sizes, and rely on diagnostic agreement or surrogate endpoints rather than clinical outcomes [38,41,42]. While some studies suggest FFR-guided revascularisation may improve outcomes, emerging evidence also indicates that NHPR may overestimate lesion severity without clear prognostic benefit [51].

An emerging alternative to wire-based physiology assessment is quantitative flow ratio (QFR), an angiography-derived index that estimates lesion-specific pressure loss from routine coronary angiographic images. QFR is particularly attractive in severe AS because it avoids both coronary pressure wire instrumentation and pharmacologically induced hyperaemia, thereby circumventing two potential limitations of FFR assessment in this population. In addition, QFR can be obtained rapidly during diagnostic angiography without prolonging procedural time substantially.

Several studies have evaluated QFR in patients with severe AS undergoing TAVR (Table 2). Across these studies, QFR has demonstrated generally good agreement with invasive FFR and NHPR measurements, with diagnostic accuracies comparable to those reported in stable CAD populations [55,56,57,58,59,60]. Importantly, some studies suggest that QFR may perform particularly well when NHPR is used as the reference standard, potentially reflecting on stability of NHPRs after valve intervention [58]. More recently Murray law-based QFR has shown encouraging diagnostic performance against ischaemia-based reference standards, further supporting the feasibility of angiography-derived physiological assessment in severe AS [55].

Despite these promising findings, the current evidence remains limited to relatively small observational studies with diagnostic validation cohorts. No studies have yet demonstrated that QFR-guided revascularisation improves clinical outcomes in this cohort. Accordingly, QFR should currently be viewed as an emerging and potentially valuable adjunctive tool rather than a replacement for established physiological assessment strategies.

Interpretation of the available literature requires recognition of important methodological limitations. The evidence base is characterised by substantial heterogeneity in study design, patient selection, physiological reference standards and clinical endpoints. Most studies are observational, involve relatively small sample sizes and focus primarily on diagnostic performance or physiological surrogate measures rather than hard outcomes. Consequently, formal evidence-grading frameworks such as GRADE are difficult to apply meaningfully within the context of this narrative review, and current conclusions should be viewed as hypothesis-generating rather than definitive. In summary, no single invasive or wire-free index can be considered definitive in this population. Instead, physiological assessment should be interpreted within the broader clinical context, recognising the limitations of each modality and the altered haemodynamic environment in severe AS.

## 4. CT Coronary Angiography-Derived Haemodynamic Assessment

### 4.1. Feasibility

While invasive physiological studies can be challenging to use, there is an unmet need to develop a non-invasive modality to assess the functional significance of CAD in the setting of severe AS. CT-derived FFR (CTFFR) has emerged as a non-invasive technology for functional assessment of coronary lesions using standard CT coronary angiography datasets [61]. In patients undergoing TAVR workup, gated CT imaging is routinely performed for annular measurements and visualisation of the ascending aorta and peripheries for suitability for vascular access, making CTFFR an attractive adjunct without requiring additional invasive procedures or pharmacological hyperaemia [62]. CTFFR also has its limitations in this cohort as these patients often have heavily calcified arteries or would have had prior surgical or percutaneous revascularisation, making applicability of this technology limited.

### 4.2. Diagnostic Performance

Table 3 summarises the current literature on the use of CTFFR in the setting of severe AS.

Ten studies in the literature have thus far investigated the use of CTFFR in the setting of severe aortic stenosis. Across available studies, CTFFR demonstrates moderate diagnostic accuracy when compared with invasive FFR [65], with reported accuracies ranging from 70 to 85% [65,66,67,68,70,71]. These findings are encouraging given the technical challenges associated with this population, including extensive coronary calcification, prior coronary revascularisation and image artefacts, all of which are common in patients with severe AS.

However, important limitations should be recognised. Gohmann et al. suggest that if CTFFR is used unselectively, it may increase false-positive CAD diagnoses [62], potentially leading to unnecessary invasive angiography. This highlights the appropriate patient selection and integration of CTFFR within a broader diagnostic framework rather than a standalone decision-making tool. By combining it with other methods, i.e., TAVR-CT [66,68] or CAD-RADS classification [67], the accuracy can be enhanced, thus reducing the need for invasive tests. These studies used an onsite machine learning-based workstation rather than a remote processing system.

A consistent strength across studies has been the high negative predictive value of CTFFR, reported between 83% and 96%. This characteristic supports its potential role as a gatekeeper strategy to safely exclude physiologically significant CAD and reduce unnecessary invasive angiography in selected patients undergoing TAVR workup.

### 4.3. Prognostic Implications

Emerging data suggest that CTFFR may provide incremental prognostic information, with associations reported between abnormal CTFFR and adverse cardiovascular outcomes following TAVR [66]. However, these findings are based on observational studies and have not been consistently validated across larger cohorts. Overall, the current evidence is limited and somewhat conflicting, highlighting the need for further prospective studies incorporating invasive physiological validation.

### 4.4. Clinical Application and Limitations

CTFFR may be most useful as a gatekeeper to invasive angiography, particularly in patients with intermediate (50–70%) stenoses on CT coronary angiography. Its high negative predictive value supports its role in safely deferring invasive assessment in selected patients. However, limited availability of onsite processing, reliance on offsite computational platforms and variability in diagnostic performance currently restrict widespread clinical adoption. More broadly, CTFFR aligns with the emerging paradigm of imaging-guided cardiovascular prevention, whereby anatomical and functional information obtained from a single non-invasive examination can support risk stratification, guide management decisions and potentially reduce unnecessary testing [72].

In summary, CTFFR represents one of the most promising non-invasive physiological approaches in severe AS, particularly as a gatekeeper strategy, although prospective validation against clinical outcomes remains necessary before routine adoption.

## 5. Proposed Management Algorithm and Perspectives Based on Expert Opinion

The available data from small cohort studies in invasive and non-invasive studies demonstrate that assessing the functional significance of CAD in patients with severe AS undergoing TAVR continues to remain a challenge. Its association with predicting adverse clinical outcomes has not been demonstrated in the current body of evidence. The results from the review suggest that NHPRs are more stable invasive metrics than FFR in the presence of severe AS and have lesser changes post valve intervention [16,17,35,43,44,45,46], whereas FFR demonstrates unpredicted variability pre and post valve intervention [41,42,45]. The non-invasive evaluation with CTFFR is a feasible alternative and shows promise. The optimal ischaemic cutoff values remain unresolved for both FFR and NHPRs. Several diagnostic studies, including a recent prospective study, have suggested that a higher FFR threshold (approximately ≤0.83) and a lower NHPR threshold (approximately ≤0.85) may more accurately predict ischaemia in patients with severe AS [52], but more studies are required to validate these findings. While these findings are biologically plausible and consistent with the known coronary alterations in coronary haemodynamics associated with AS, they remain exploratory. The proposed thresholds have not yet been validated against hard clinical outcomes and should therefore be viewed as hypothesis-generating rather than being used as definitive decision thresholds for revascularisation.

**Figure 2:**
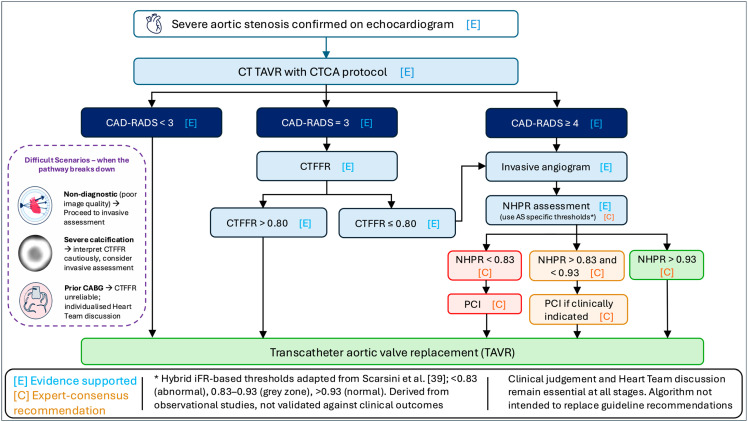
**Central Illustration:** Proposed management framework of patients with coronary artery disease and severe aortic stenosis. CTCA—CT coronary angiography; CTFFR—CT-derived fractional flow reserve; NHPR—Non-hyperaemic pressure ratio; PCI—percutaneous coronary intervention; CAD-RADS—Coronary artery disease reporting and data system.

It would be ideally convenient if CT could be utilised as a one-stop solution for anatomical and functional evaluation of patients undergoing TAVR [73], but there remains a lack of empirical evidence to recommend CTFFR over invasive metrics. The results from the DEPICT-CTA collaboration have demonstrated that routine CT-TAVR protocol provides high diagnostic accuracy to rule out left main and proximal coronary artery stenosis using a threshold of up to 70% diameter stenosis in avoiding an invasive coronary angiogram in 70% of patients [73]. Brandt et al. have demonstrated that the CAD-RADS system of classification is also a sensitive screening tool to determine which lesions need further haemodynamic assessment [67]. Thus, in patients with moderate coronary artery disease (50–69%) on CT, further evaluation with CTFFR would be a reasonable approach before embarking on an invasive angiogram. Due to the drawbacks of invasive and CT-derived FFR in severe AS described above, we propose a traffic signal approach incorporating a hybrid assessment by both methods to confirm functional significance (Figure 2). The NHPR thresholds incorporated within the Central Illustration are adapted from Scarsini et al. [39], which uses an iFR < 0.83 to identify lesions likely to warrant treatment and iFR > 0.93 to support deferral. These thresholds are different from the NHPR ≤ 0.85 discussed earlier, which was derived from studies evaluating diagnostic performance against ischaemia reference standards. Both approaches remain exploratory and have not been prospectively validated against clinical outcomes in severe AS. This proposed approach represents an expert-informed synthesis of available evidence rather than a guideline-endorsed strategy.

CT coronary angiography can serve as an initial screening tool, allowing a proportion of patients to proceed directly to TAVR without invasive angiography. In patients with moderate lesions, CTFFR may provide a pragmatic and physiologically informed strategy for clinical decision-making. Applying the traditional cutoff of >0.80 as a defer strategy and ≤0.80 to prompt further workup with an invasive angiogram allows clinicians to selectively triage patients who are most likely to benefit from coronary intervention. Such an approach has the potential to reduce unnecessary invasive procedures, limiting procedural risk and resource utilisation while preserving access to revascularisation for those with haemodynamically significant lesions. In this context, CTFFR serves as an effective gatekeeper to the invasive catheterisation laboratory and may translate to improved patient-centred outcomes.

Importantly, the negative predictive value of CTFFR has been reported to be between 83 and 96% [65,74,75] irrespective of the degree of calcification. While these data support its reliability in ruling out functionally significant stenoses, they also indicate that a small proportion of ischaemic lesions may remain undetected when CTFFR is used as a standalone defer strategy. This limitation underscores the need for careful clinical integration of imaging findings with symptom, risk profile, and other diagnostic data.

Given the limitations of both invasive and non-invasive modalities, a hybrid approach using CT and invasive metrics may provide a robust physiological assessment for risk stratification and management. By integrating invasive and non-invasive metrics, this model may optimise identification of lesions that confer future cardiovascular risk. Further validation in large prospective studies is warranted to clarify long-term prognostic impact and refine patient selection criteria. More broadly, the haemodynamic consequences of severe AS extend beyond the coronary circulation, contributing to multisystem vulnerability including cardiorenal dysfunction and peri-procedural organ injury, reinforcing the importance of a holistic assessment strategy when evaluating patients undergoing TAVR [76].

## 6. Revascularisation of Concomitant Coronary Artery Disease

### 6.1. Identifying Significant Lesions

Identification and quantification of functional significance of coronary lesion in severe AS and demonstrating benefit from coronary revascularisation are related but distinct clinical questions. Physiological indices such as FFR and NHPRs are designed to identify lesions capable of limiting coronary flow and produce myocardial ischaemia. However, the ability to diagnose ischaemia does not necessarily imply that the treatment of the identified lesion will improve symptoms or improve survival [24]. This distinction is particularly relevant in severe AS, where physiological thresholds remain uncertain and the interaction between valvular stenosis and coronary disease complicates attribution of symptoms and prognosis.

As discussed, both FFR and NHPR are affected in opposing directions, leading to uncertainty regarding optimal thresholds. While invasive physiology provides additional information beyond angiography alone, its interpretation must be individualised and reliance on standard cutoffs may be inappropriate.

### 6.2. Does Revascularisation Improve Outcomes?

The question of whether treating CAD in severe AS improves clinical outcomes is distinct from identifying lesion significance. The benefit of treating concomitant CAD in severe AS remains speculative, and various randomised controlled trials are currently testing this hypothesis. The ACTIVATION trial [77] was one such trial, which demonstrated no clear benefit of routine PCI prior to TAVR using an angiography-guided strategy. Untreated CAD in AS confers an increased cardiovascular mortality at 12 months, especially in patients with complex multivessel CAD [78,79]. Secondly, the development of safe methods of coronary intervention through a TAVR device will be essential in the forthcoming era of TAVR in younger and lower-risk patients. This strengthens the case for physiological assessment of CAD, at least in the younger population awaiting TAVR.

Caution is warranted in several clinically important subgroups where both physiological assessment and revascularisation decision-making become increasingly complex. Patients with multivessel disease frequently exhibit diffuse atherosclerosis, serial lesions and competing sources of ischaemia, all of which may complicate interpretation of lesion-specific physiology. Similarly, patients with reduced left ventricular ejection fraction may demonstrate altered coronary haemodynamics and symptom profiles that are not solely attributable to epicardial coronary stenoses. Advanced extra-valvular cardiac damage, including severe LV hypertrophy, myocardial fibrosis, pulmonary hypertension, right ventricular dysfunction and significant concomitant valvular disease, may further confound attribution of symptoms and prognosis to individual coronary lesions. In these settings, the relationship between physiological lesion significance, symptom burden and clinical outcomes is likely to be more complex and the potential benefits of PCI remain less certain. Consequently, physiological measurements should be interpreted with the broader clinical context and integrated with imaging findings, patient characteristics and multidisciplinary Heart Team discussion.

Recent randomised trials have provided encouraging evidence supporting physiology-guided revascularisation strategies in patients undergoing TAVR. In FAITAVI and the evidence from Lonborg et al. [80,81], physiology-guided PCI was associated with improved clinical outcomes compared with angiography-guided management strategies. Importantly, these studies used the conventional FFR threshold of ≤0.80 to define lesion significance. This creates an important conceptual tension with the current evidence base. While several diagnostic studies in severe AS suggest that higher FFR thresholds (approximately ≤0.83) [17,43,45] and lower NHPR thresholds (approximately (≤0.85)) [16,17,35,43,44,45,46,55] may more accurately identify ischaemia, outcome data supporting revascularisation are currently linked to traditional FFR thresholds rather than these proposed AS-specific cutoffs.

Accordingly, it cannot be assumed that lesions identified by the AS-specific thresholds will derive the same benefit from revascularisation as lesions meeting conventional FFR and NHPR criteria. Conversely, the thresholds that appear to best predict ischaemia may not yet be the thresholds supported by outcome-based evidence. This distinction highlights the need for prospective studies linking physiological measurements, revascularisation decisions and clinical outcomes before these modified threshold criteria can be adopted in routine clinical practice. Optimal Timing of Transcatheter Aortic Valve Implantation and Percutaneous Coronary Intervention [TAVI PCI]; NCT04310046 is a physiology-guided revascularisation trial in TAVR which is currently recruiting. The results of this trial will shed further light on the best strategies for managing CAD in severe AS. Pending these data, it will be reasonable to consider the proposed hybrid functional evaluation in younger patients awaiting TAVR.

## 7. Future Research Priorities

Several key uncertainties remain in the assessment and management of CAD in severe AS. The next phase of research in coronary physiology for severe AS should move beyond diagnostic agreement studies towards prospective outcome-driven trials. Several key questions can be prioritised according to their likely clinical impact.

### 7.1. Priority 1: Validation of Thresholds Against Clinical Outcomes

The highest priority is prospective validation of proposed as-specific thresholds. Several studies suggest that FFR thresholds around ≤0.83 and NHPR thresholds around ≤0.85 may more accurately identify myocardial ischaemia in severe AS than conventional cutoffs. These studies should determine whether lesions identified by the modified cutoffs translate to meaningful clinical outcomes, including death, myocardial infarction, unplanned revascularisation and symptom burden. Until these studies are available, these thresholds should remain hypothesis-generating.

### 7.2. Priority 2: Determining Whether Improved Diagnosis Translates into Improved Outcomes

A critical question is whether physiological strategies that appear diagnostically superior ultimately improve patient outcomes. Future randomised trials should compare revascularisation strategies using conventional thresholds versus the proposed AS-specific thresholds. Importantly, outcome measures should go beyond diagnostic concordance and include hard clinical endpoints. Patient-centred outcomes including angina relief, quality of life and functional status should also be incorporated.

### 7.3. Priority 3: Defining the Role of Non-Wire-Based Physiological Assessment

CTFFR and QFR represent attractive non-wire-based alternatives to conventional wire-based physiology assessment. Future studies should evaluate whether these strategies can safely guide revascularisation without compromising clinical outcomes. Focus should be placed on validating their performance in patients with severe calcification and prior coronary intervention, populations that remain underrepresented in existing studies.

Overall, future research should prioritise outcome-based validation over further diagnostic concordance studies. Demonstrating that physiological assessment changes clinical outcomes, rather than simply improving agreement with surrogate measures of ischaemia, represents the most important remaining challenge in this field.

## 8. Conclusions

In patients with severe AS, coronary physiology is fundamentally altered, limiting the direct application of conventional ischaemic thresholds. Both FFR and NHPR provide useful but imperfect assessments, with frequent discordance reflecting underlying haemodynamic complexity rather than a measurement error. CT-based approaches offer a promising non-invasive strategy, particularly as a gatekeeper to invasive angiography.

At present, an individualised multimodality approach integrating anatomical, physiological and CT-derived information appears most appropriate, recognising that proposed severe AS-specific physiological thresholds remain hypothesis-generating and require prospective outcome validation.

## Figures and Tables

**Table 1:** Summary of the published literature describing invasive epicardial physiological indices (FFR and NHPR) in severe AS.

Authors	Citation	Study Design and Hypothesis	Number of Patients	Number of Coronary Lesions	Conclusions
Ahmad et al. [16]	*JACC: Cardiovascular Interventions* 2018	Prospective, multicentre observational study. To assess the effects of treating AS on FFR, iFR and coronary flow.	28	30	iFR did not change post TAVR (0.88 vs. 0.88, *p* = 0.73), FFR decreased significantly post TAVR (0.87 vs. 0.85, *p* = 0.001).
Comella et al. [17]	*Cardiovascular Revascularization Medicine*, 2022	Prospective observational study. To assess agreement between iFR and other non-hyperaemic pressure ratios.	42	67	iFR and the other NHPR have good correlation with FFR but have a lower optimal cutoff than non-AS patients.
Vendrik et al. [35]	*Journal of American Heart Association*, 2020	Prospective, observational study. To determine effects of TAVR on coronary blood flow and coronary physiology.	13	-	TAVR acutely improves whole-cycle hyperaemic coronary flow with ongoing improvements at 6 months follow-up, ongoing decrease in FFR at 6 months; conversely, no change in iFR on serial measurements.
Lunardi et al. [38]	*Journal of American Heart Association* 2019	Retrospective analysis of prospective registry. Angiographic-guided v physiology-guided (FFR cutoff value—0.8) revascularisation in patients undergoing TAVR.	122 in Angiography arm and 94 in FFR arm	184 in Angiography arm and 142 in FFR arm	FFR-guided group had better MACE-free survival as compared with Angio-guided (92.6% vs. 82%, HR 0.4, *p* = 0.035).
Scarsini et al. [39]	*Cardiovascular Revascularization Medicine*, 2018	Prospective, observational study. To assess the hybrid real-time iFR-FFR approach to physiologically evaluate CAD in severe AS.	62	141	A “defer” iFR value of >0.93 yielded an NPV of 98.4% to exclude FFR non-significant lesions and a “treatment” iFR value of <0.83 had a PPV of 91.3% to identify FFR significant lesions.
Scarsini et al. [40]	*International Journal of Cardiology*, 2017	Prospective observational study. To compare iFR and FFR in patients with severe AS and CAD.	85	179	Conventional iFR cutoff, 0.89, had lower diagnostic agreement with FFR. A lower threshold of 0.83 had better agreement with FFR < 0.8.
Stundl et al. [41]	*Clinical Research in Cardiology*, 2020	Prospective observational. To detect changes in haemodynamic significance of coronary lesions post TAVR and explore outcomes of FFR-positive CAD.	131 AS with CAD; 31 had pre-TAVR FFR	38 lesions in 31 patients	No significant difference in FFR values pre and post TAVR and no significant event in the follow-up period.
Pesarini et al. [42]	*Circulation Cardiovascular Interventions*, 2016	Prospective observational study. To assess changes in FFR values immediately pre and post TAVR as well as 30-day outcomes.	54 patients	133 lesions	No significant overall change in FFR (0.89 vs. 0.89, *p* = 0.73). No patient with FFR positive at baseline had clinical events at time of procedure and at 30 days.
Kleczynski et al. [43]	*Advances in Medical Sciences*, 2021	Prospective registry. To assess diagnostic agreement between FFR and iFR in the setting of severe AS.	221	416	FFR and iFR have a good diagnostic agreement (ICC 0.83), with lower optimal thresholds than non-AS patients.
Arashi et al. [44]	*Cardiovascular Intervention and Therapeutics*, 2019	Retrospective study. To compare utility of iFR in severe AS using FFR as a comparison.	158	217	iFR showed good correlation with FFR in AS. Cutoff value for iFR was 0.73 to predict FFR < 0.8.
Scarsini et al. [45]	*International Journal of Cardiology*, 2020	Prospective observational pilot study. To assess long-term variations in FFR and iFR after TAVR.	14	23	FFR remained stable if clearly normal (>0.85) but decreased if borderline or abnormal at baseline. iFR did not show a trend at long term post TAVR.
Scarsini et al. [46]	*EuroIntervention*, 2018	Prospective observational study. To assess changes in iFR values before and after TAVR in severe AS and CAD.	66	145	iFR values did not change overall but showed significant and erratic individual variations after valve replacement. The diagnostic accuracy of iFR in predicting FFR < 0.8 was poor (65%).
Ahmad et al. [47]	*Circulation: Cardiovascular Interventions*, 2019	Prospective observational study. To quantify the effects of severe AS on coronary microcirculation and determine if this is influenced by concomitant CAD. This was then compared to the effect of coronary stenoses on coronary microcirculation.	55 patients with severe AS and CAD compared with 85 patients with CAD but no AS	-	TAVR improves microcirculatory function regardless of coronary lesion severity and this improvement is equivalent to haemodynamic benefit of stenting coronary lesions with iFR < 0.74.
Comella et al. [48]	*JACC: Cardiovascular Interventions*, 2021	Prospective, observational study. To assess the discordance between FFR and NHPRs in patients with severe AS.	41	-	There is discordance between FFR and NHPR in 20% patients, with a very distinct pattern being FFR−/NHPR+.
Stoller et al. [49]	*EuroIntervention*, 2018	Prospective observational study. To assess changes in coronary haemodynamics with LV afterload reduction in severe AS by TAVR.	40 patients with severe AS, of which 26 had CAD and 14 no CAD.	-	CFR does not appear to be acutely affected by TAVR, but there is an improvement in FFR.
Yamanaka et al. [50]	*Journal of Cardiology*, 2022	Retrospective cohort study. To assess discordance between FFR and iFR in severe AS.	140	164	There is discordance between FFR and iFR in 29% patients and predominantly FFR−/iFR+.
Jo et al. [51]	*Circulation: Cardiovascular Interventions*, 2024	Retrospective analysis of FFR and iFR in patients with and without severe AS.	293 with severe AS and 1882 without	395 lesions in severe AS and 2257 without	FFR is less affected by severe AS and is associated with prognosis, iFR may overestimate functional severity without prognostic significance.
Minten et al. [52]	*JACC: Cardiovascular Interventions*, 2025	Prospective analysis to assess relationship between FFR and RFR, long-term changes post AVR on these indices and to determine ischaemic cutoffs.	116	146	There was a discordance of 42% (FFR−/RFR+) at baseline. Six months after AVR, FFR decreases and RFR increases significantly (−0.03 vs +0.04, *p* < 0.0001 for both). Best ischaemic cutoffs FFR ≤ 0.83 and RFR ≤ 0.85.
Fezzi et al. [53]	*International Journal of Cardiology*, 2025	To determine predominant physiological pattern of coronary disease in severe AS and to assess the impact of TAVR on pre and post FFR and iFR.	67	136	Diffuse CAD without major gradients was the predominant physiological pattern. Post TAVR, FFR decreased in vessels with major focal gradients, while iFR changes were more unpredictable.
Dziewierz et al. [54]	*Cardiovascular Revascularization Medicine*, 2025	To identify angiographic predictors of FFR/iFR discordance and define angiographic phenotypes using machine learning.	221	401	FFR/iFR discordance occurred in 7.5% lesions with %diameter stenosis being the only independent predictor of discordance. The discordance manifests solely as FFR−/iFR+ pattern.

FFR—fractional flow reserve; MACE—major adverse cardiovascular event; iFR—instantaneous wave-free ratio; TAVR—transcatheter aortic valve replacement; CAD—coronary artery disease; RFR—resting full cycle ratio; CFR—coronary flow reserve; NHPR—non-hyperaemic pressure ratio; AS—aortic stenosis; NPV—negative predictive value; PPV—positive predictive value; AVR—aortic valve replacement; ICC—intra-class coefficient.

**Table 2:** Studies evaluating QFR in severe aortic stenosis.

Authors	Citation	Study Design and Hypothesis	Number of Patients	Number of Coronary Legions	Conclusions
Yuta et al. [55]	*Heart and Vessels*, 2024	Prospective analysis to evaluate the diagnostic performance of pre-TAVR QFR, μQFR and iFR using post-TAVR FFR ≤ 0.80 as reference.	25	38	μQFR significantly correlated with post-TAVR FFR (r = 0.73, *p* < 0.001) with an accuracy, sensitivity, specificity, PPV and NPV of 84.2%, 61.6%, 96%, 88.9% and 82.8%. Best cutoff value was 0.88. For iFR they were 76.5%, 90.9%, 69.6%, 58.8% and 94.1% respectively. Best cutoff value was 0.89. For QFR they were 81/5%, 69.2%, 88%, 75% and 84.6%. Best cutoff value was 0.91.
Sejr-Hansen et al. [56]	*Catheter Cardiovascular Interventions*, 2022	Retrospective, multicentre, investigator-initiated study. To assess diagnostic performance of pre-TAVR QFR using post-TAVR FFR and post-TAVR iFR as references.	28	29	Pre-TAVR QFR showed a good diagnostic performance using post-TAVR FFR as reference (83%) as compared with post-TAVR iFR (52%); *p* = 0.008.
Mejia-Renteria et al. [57]	*EuroIntervention*, 2020	Retrospective, international, multicentre study. To assess diagnostic performance of QFR as compared with FFR in severe AS and CAD.	115	138	Compared with FFR, QFR has good diagnostic yield and is superior to angiography alone.
Kleczynski et al. [58]	*Rev Esp Cardiol*, 2021	Prospective observational study. To assess diagnostic performance of QFR with Pd/PA, FFR and iFR.	221	416	QFR had good agreement with FFR. However, its diagnostic accuracy was better when iFR was used as the reference.
Zasada et al. [59]	*Advances in Interventional Cardiology*, 2022	Prospective, observational study. To compare FFR, iFR and QFR in intermediate lesions in severe AS.	12	13	There was 100% agreement between FFR and iFR. Agreement between FFR/iFR with QFR was 69%.
Dowling et al. [60]	*Cardiovascular Diagnosis and Therapy*, 2022	Prospective, observational study. To determine the diagnostic accuracy of QFR in severe AS.	35	57	QFR demonstrated acceptable diagnostic performance as compared with FFR (AUC 0.92), iFR (AUC 0.92), dPR (AUC 0.90) and Pd/Pa (AUC 0.89).

TAVR—transcatheter aortic valve replacement; QFR—quantitative flow ratio; iFR—instantaneous wave-free ratio; FFR—fractional flow reserve; Pd/Pa—pressure distal/pressure aortic; AUC—area under the curve; dPR—diastolic pressure ratio; AS—aortic stenosis; CAD—coronary artery disease.

**Table 3:** Summary of studies describing use of CTFFR in severe AS.

Authors	Citation	Study Design and Hypothesis	Number of Patients	Number of Coronary Lesions	Conclusions
Gohmann et al. [62]	*Journal of Clinical Medicine*, 2020	Prospective study to evaluate the ability of onsite CT-FFR to correctly classify cases without significant CAD on CCTA as compared to invasive angiogram.	109	436	Unselectively applied, CT-FFR may vastly increase the number of false-positive ratings of CAD compared to morphological scoring.
Zhang et al. [63]	*European Radiology*, 2021	Retrospective analysis. Pre TAVR-CT used to calculate CT-FFR. The aim was to evaluate the impact of TAVR on CT-FFR values.	190 each pre and post TAVR, 80 at 1-year mark	568 each pre and post TAVR, 243 at 1-year mark	TAVR improves CT-FFR values in patients with compromised coronary blood flow.
Michiels et al. [64]	*International Journal of Cardiovascular Imaging*, 2021	Prospective study to assess the effects of SAVR or TAVR on CT-FFR.	25	75	CT-FFR is not subject to the confounding effect of LV mass regression after SAVR or TAVR. Despite significant LV mass regression at 6 months after SAVR or TAVR, CT-FFR values remained constant.
Michail et al. [65]	*Circulation: Cardiovascular Interventions*, 2021	Prospective evaluation of safety, feasibility and validity of offsite CT-FFR in severe AS.	39	60	CT-FFR is safe and feasible with a sensitivity, specificity, PPV and NPV of 73.9%, 78.4%, 68% and 82.9%, and an accuracy of 76.7%.
Aquino et al. [66]	*Radiology*, 2022	Retrospective study to evaluate the predictive value of onsite CT-FFR for adverse clinical outcomes in TAVR patients.	196	-	CT-FFR was associated with MACE and improved the predictive value of coronary CT angiography assessment.
Brandt et al. [67]	*European Radiology*, 2022	Retrospective analysis to evaluate feasibility and diagnostic performance of onsite CT-FFR for detection of significant CAD and decision-making in patient with severe AS, to potentially avoid additional invasive angiogram.	95	-	Combination of CT-FFR and CAD-RADS can identify significant CAD pre-TAVR with a sensitivity, specificity, PPV and NPV of 100%, 78%, 40% and 100% respectively, potentially decreasing the number of ICAs by 68%.
Peper et al. [68]	*JACC: Cardiovascular Interventions*, 2022	Retrospective analysis to assess the diagnostic performance of onsite CT-FFR for the diagnosis of CAD pre TAVR.	338	977	CT-FFR significantly improves the diagnostic accuracy of CCTA is diagnosing significant CAD on ICA with a sensitivity, specificity, PPV and NPV of 84.6%, 88.3%, 63.2% and 96% with a diagnostic accuracy of 87.6% on a per patient level.
Steyer et al. [69]	*Radiology: Cardiothoracic Imaging*, 2024	Retrospective evaluation to examine clinical feasibility of workstation-based CT-FFR system to evaluate CAD and predict MACE within 24 months post TAVR.	112	-	Compared with conventional CAD markers, CT-FFR better predicted adverse outcomes after TAVR.
Sasaki et al. [70]	*Circulation Journal*, 2024	Prospective analysis to evaluate diagnostic performance of pre-TAVR FFRCT and iFR to predict post-TAVR FFR ≤ 0.8.	21	34	The diagnostic accuracy of CTFFR to predict post-TAVR FFR ≤ 0.8 is 82% with a sensitivity, specificity, PPV and NPV of 83%, 82%, 71% and 90%. The optimal pre-TAVR CTFFR was 0.78 and pre-TAVR iFR was 0.89 to predict post-TAVR FFR ≤ 0.8.
Thakkar et al. [71]	*Journal of Cardiovascular Computed Tomography*, 2025	To assess the feasibility and diagnostic accuracy of an onsite workstation to detect FFR ≤ 0.8 in patients with severe AS.	38	59	There was moderate correlation between CTFFR and FFR (r = 0.65, *p* < 0.001) with Bland–Altman analysis indicating a mean bias ± SD of 0.04 ± 0.12. Sensitivity, specificity, positive and negative predictive values of 79%, 62%, 79%, and 62%, with an accuracy of 73% and AUC of 0.79.

FFR—fractional flow reserve; AS—aortic stenosis; CTFFR—CT-derived fractional flow reserve; CAD—coronary artery disease; TAVR—transcatheter aortic valve replacement; iFR—instantaneous wave-free ratio; MACE—major adverse cardiovascular event; CAD-RADS—coronary artery disease reporting and data system.

## Data Availability

No new data were created.
